# Synchronicity and Rhythmicity of Purkinje Cell Firing during Generalized Spike-and-Wave Discharges in a Natural Mouse Model of Absence Epilepsy

**DOI:** 10.3389/fncel.2017.00346

**Published:** 2017-10-31

**Authors:** Lieke Kros, Sander Lindeman, Oscar H. J. Eelkman Rooda, Pavithra Murugesan, Lorenzo Bina, Laurens W. J. Bosman, Chris I. De Zeeuw, Freek E. Hoebeek

**Affiliations:** ^1^Department of Neuroscience, Erasmus MC, Rotterdam, Netherlands; ^2^Department of Neurosurgery, Erasmus MC, Rotterdam, Netherlands; ^3^Netherlands Institute for Neuroscience, Royal Dutch Academy for Arts and Sciences, Amsterdam, Netherlands

**Keywords:** epilepsy, cerebellum, inferior olive, tottering, oscillations

## Abstract

Absence epilepsy is characterized by the occurrence of generalized spike and wave discharges (GSWDs) in electrocorticographical (ECoG) recordings representing oscillatory activity in thalamocortical networks. The oscillatory nature of GSWDs has been shown to be reflected in the simple spike activity of cerebellar Purkinje cells and in the activity of their target neurons in the cerebellar nuclei, but it is unclear to what extent complex spike activity is implicated in generalized epilepsy. Purkinje cell complex spike firing is elicited by climbing fiber activation and reflects action potential firing in the inferior olive. Here, we investigated to what extent modulation of complex spike firing is reflected in the temporal patterns of seizures. Extracellular single-unit recordings in awake, head-restrained homozygous *tottering* mice, which suffer from a mutation in the voltage-gated Ca_V_2.1 calcium channel, revealed that a substantial proportion of Purkinje cells (26%) showed increased complex spike activity and rhythmicity during GSWDs. Moreover, Purkinje cells, recorded either electrophysiologically or by using Ca^2+^-imaging, showed a significant increase in complex spike synchronicity for both adjacent and remote Purkinje cells during ictal events. These seizure-related changes in firing frequency, rhythmicity and synchronicity were most prominent in the lateral cerebellum, a region known to receive cerebral input via the inferior olive. These data indicate profound and widespread changes in olivary firing that are most likely induced by seizure-related activity changes in the thalamocortical network, thereby highlighting the possibility that olivary neurons can compensate for pathological brain-state changes by dampening oscillations.

## Introduction

Absence epilepsy is a common form of generalized epilepsy that clinically manifests itself as behavioral arrest and diminished consciousness (Snead, 1995; Andermann and Berkovic, 2001; Norden and Blumenfeld, 2002; Fisher et al., 2014). It is characterized by thalamocortical network oscillations occurring as generalized spike and wave discharges (GSWDs) in electrocorticographical (ECoG) recordings. GSWDs appear as repetitive sharp peaks followed by slow waves reflecting synchronous phasic firing of thalamocortical and corticothalamic neurons (Snead, 1995; Andermann and Berkovic, 2001; Sorokin et al., 2017). Recruitment of inhibitory reticular thalamic neurons by either of these two cell populations has been shown to be necessary and sufficient for GSWD appearance due to their control of rhythmic burst firing of thalamic relay cells (Clemente-Perez et al., 2017; Sorokin et al., 2017). Apart from the well-studied thalamocortical pathophysiology, generalized epilepsy also involves subcortical structures (Norden and Blumenfeld, 2002). One of these subcortical structures that has been shown to be involved in pathological thalamocortical oscillations is the cerebellum (Kros et al., 2015b). Cerebellar nuclei neurons divergently project to neurons throughout the thalamic complex and a proportion of cerebellar nuclei neurons shows firing that is synchronized with thalamocortical network activity (Kandel and Buzsáki, 1993; Kros et al., 2015b). Likewise the simple spike activity of Purkinje cells in the cerebellar cortex can also be phase-locked to the GSWDs (Kandel and Buzsáki, 1993; Kros et al., 2015b).

Thalamocortical information can be conveyed to the cerebellum through two pathways: via mossy fibers that predominantly originate from pontine nuclei, and climbing fibers that exclusively originate from the inferior olive (Eccles, 1969; Simpson et al., 1996; Apps and Garwicz, 2005). These afferent pathways both project to the cerebellar cortex and send collaterals to the cerebellar nuclei (Chan-Palay, 1975; Groenewegen et al., 1979; Van der Want et al., 1989; Gauck and Jaeger, 2003; Uusisaari and Knopfel, 2011). Typically, cerebellar nuclei neurons fire at frequencies of 20–100 Hz in awake rodents (Hoebeek et al., 2010; De Zeeuw et al., 2011; Person and Raman, 2012). Given that the intrinsic spiking activity of cerebellar nuclei neurons has been shown to be regular (Jahnsen, 1986; Mouginot and Gähwiler, 1995), the GSWD-related modulation may be attributed at least in part to synaptic inputs (Kros et al., 2015a). Although cerebellar nuclei neurons are known to receive both excitatory and inhibitory input from various intra- and extra-cerebellar sources, previous studies indicate that the most potent source of cerebellar nuclei firing modulation occurs from the GABAergic Purkinje cells (Gauck and Jaeger, 2003; Alviña et al., 2008; De Zeeuw et al., 2008; Hoebeek et al., 2010; Person and Raman, 2012; ten Brinke et al., 2015; Dykstra et al., 2016; Lu et al., 2016). In rodents, ~30–50 Purkinje cell axons converge on a single cerebellar nuclei neuron (Sugihara et al., 2009; Person and Raman, 2012). Thus, the simple spike modulation of Purkinje cells being phase-locked to the GSWDs (Kandel and Buzsáki, 1993) might also contribute to the generation and/or control of the seizure. However, to what extent the Purkinje cell simple spike modulation stands on its own and what the impact of oscillatory complex spike activity is, remains to be elucidated.

Purkinje cell simple spike firing is commanded by intrinsic pacemaking activity and mossy fiber input, which is forwarded by granule cells and molecular layer interneurons, whereas complex spike firing exclusively reflects climbing fiber activation generated in the inferior olive. Given that (i) the axons of single olivary neurons branch and synapse in ~7 Purkinje cell dendritic trees (Van der Want et al., 1989), (ii) olivary neurons tend to synchronize their membrane depolarizations (Devor and Yarom, 2002), and (iii) synchronous olivary activity is a potent way of controlling firing patterns of cerebellar nuclei neurons (as reviewed by De Zeeuw et al., 2011), olivo-cerebellar projections appear to be suitable candidates to mediate the oscillatory firing patterns from the cerebral cortex to the cerebellum. Indeed, olivary lesions have been shown to have anti-epileptic effects in epilepsy-prone rats (Welsh et al., 1998) and patients with dentate-olivary dysplasia are reported to suffer from intractable seizures (Harding and Boyd, 1991; Saito et al., 2006).

To establish to what extent activity of the inferior olive contributes to the oscillatory patterns of cerebellar activity during absence seizures, we studied the frequency, rhythmicity and synchronicity of Purkinje cell complex spike firing during episodes of GSWDs. We used a well characterized mouse model for absence epilepsy, the *tottering* mouse, which is characterized by a loss-of-function mutation in the *Cacna1a* gene that codes for the α1-subunit of Ca_V_2.1-channels (Noebels and Sidman, 1979; Fletcher et al., 1996). We first investigated single-unit Purkinje cell firing patterns during GSWDs by simultaneously recording ECoG in primary motor and somatosensory cortices to detect any change in firing frequency and subsequently used extracellular multiple-unit Purkinje cell recordings and Ca^2+^-imaging in Purkinje cell dendrites in awake, head-restrained homozygous *tottering* mice to study synchronicity.

## Materials and methods

All experiments were performed in accordance with the European Communities Council Directive. Protocols were reviewed and approved by an independent animal ethical committee (DEC Consult, Soest, Netherlands).

### Animals

Data were collected from male and female homozygous *tottering* mice (4–30 weeks old) and their wild-type littermates, which were bred using heterozygous mice. As described previously (Kros et al., 2015b) the colony, originally purchased from Jackson laboratory (Bar Harbor, ME, USA), was maintained using C57BL/6NHsd mice obtained from Envigo laboratories (Horst, Netherlands). PCR was used to confirm the presence of the *tottering* mutation in the *Cacna1a* gene using 5′-TTCTGGGTACCAGATACAGG-3′ (forward) and 5′-AAGTGTCGAAGTTGGTGCGC-3′ (reverse) primers (Eurogentech, Seraing, Belgium) and subsequent digestion using restriction enzyme NSBI at the age of P9–P12.

### Experimental procedures

#### Surgery

Mice were anesthetized with isoflurane (4–5% for induction, 1.5–2.5% for maintenance) after which carprofen (5 mg/kg) and buprenorphine (50 μg/kg) were given systemically and lidocaine (2%) was applied subcutaneously to the skull. Hereafter, the skull was exposed, cleaned and treated with OptiBond All-In-One (Kerr Corporation; Orange, CA, USA). Subsequently, five 200 μm diameter silver ball tip electrodes constructed from teflon-coated silver wire (Advent research materials, Eynsham, Oxford, UK) or five 1 mm stainless steel screws were subdurally implanted for ECoG recordings above the primary motor cortex (+1 mm AP; ± 1 mm ML relative to bregma, bilateral), primary sensory cortex (−1 mm AP; ± 3.5 mm ML, bilateral) and in the rostral portion of the interparietal bone to serve as reference (−1 mm AP relative to lambda). Electrodes and connectors were fixed to the skull and embedded in a pedestal composed of the hybrid composite or dental acrylic (Simplex Rapid; Associated Dental Products, Kemdent works, Purton, Wiltshire, UK and Charisma; Heraeus Kulzer, Hanau, Germany, respectively). To enable cerebellar electrophysiological recordings, bilateral craniotomies (~2 mm diameter) were carefully drilled in the occipital bone. Great care was taken to leave the dura mater intact. Bupivacaine (1 mg/kg) was locally applied to the skull surrounding the craniotomies after which a dental acrylic recording chamber (Simplex rapid) was formed. The recording chamber was sealed with bone wax (Ethicon, Somerville, NJ, USA) after covering the exposed dura with tetracycline-containing ointment (Terra-cortril; Pfizer, New York, NY, USA). An hour after surgery, the mice received another dose of the analgesic carprofen (5 mg/kg) followed by another dose of buprenorphine (50 μg/kg) after 8 h. The mice were given at least 5 days to recover and then were allowed ~3 h training sessions with ECoG monitoring for 2 days prior to the day on which the single-unit electrophysiological recordings started. In case of experiments involving two-photon Ca^2+^-imaging, a head plate instead of a recording chamber was placed around the craniotomy during surgery. The craniotomy for the two-photon experiments was made on the day of the recording. This procedure was performed under isoflurane anesthesia with local analgesia using lidocaine. Recordings started at least 1 h after termination of the anesthesia.

#### Single cell recordings

Recordings were performed in awake, head-fixed animals for no longer than 4 h while their body temperature was supported using a homeothermic pad (FHC, Bowdoin, ME, USA). Custom-made, borosilicate glass capillaries (OD 1.5 mm, ID 0.86 mm; resistance 8–12 MΩ; taper length ~8 mm; tip diameter ~1 μm) (Harvard Apparatus, Holliston, MA, USA) filled with 2 M NaCl were used for electrophysiological Purkinje cell recordings. Electrodes were positioned stereotactically using an electronic pipette holder (SM7; Luigs & Neumann, Ratingen, Germany). Neurons were recorded extracellularly in both medial (vermis) and lateral (paravermis and hemisphere) areas of the cerebellar cortex, mainly from lobules VI–X. Purkinje cells were identified by the characteristic occurrence of both complex spikes and simple spikes and a minimal pause in simple spike firing following each complex spike of 10 ms. ECoGs were filtered online using a 1–100 Hz band pass filter and a 50 Hz notch filter. Single unit extracellular recordings and ECoGs were simultaneously sampled at 20 kHz (Digidata 1322A, Molecular Devices LLC, Axon Instruments, Sunnyvale, CA, USA), amplified, and stored for off-line analysis (CyberAmp and Multiclamp 700A, Molecular Devices LLC, Axon Instruments, Sunnyvale, CA, USA). ECoG traces were down sampled to 300 Hz.

#### Multi-unit recordings

Experiments involving multiple single-unit recordings were performed under the same conditions as described above. In these experiments, Purkinje cell recordings were made using glass electrodes or using quartz-coated platinum-tungsten fiber electrodes (2–5 MΩ; 80 μm outer diameter; Thomas Recording, Giessen, Germany). The former electrodes were placed using an electronic pipette holder (SM7; Luigs and Neumann) for each of the two glass electrodes. These pipette holders were mounted on their own oil-drive to position the electrodes independently in the brain. The latter electrodes were placed in a rectangular matrix (Thomas Recording) with an inter-electrode distance of 305 μm. Also these electrodes were independently positioned in the brain. Prior to the recordings using the platinum-tungsten electrodes, the dura was removed under light isoflurane anesthesia. The recordings were started at least 30 min after termination of the anesthesia. All recordings were made in crus 1, crus 2 and vermis at a minimal depth of 500 μm from the cortical surface. The signals from the glass electrodes were sampled at 20 kHz (Digidata 1322A, Axon Instruments), amplified, and stored for offline analysis (CyberAmp and Multiclamp 700A, Axon Instruments). The signals from the platinum-tungsten electrodes were sampled at 25 kHz (PZ5 NeuroDigitizer, Tucker-Davis Technologies, Alachua, FL, USA), amplified, filtered online using a 1–6,000 Hz band-pass filter and stored offline using a RZ2 multi-channel workstation (Tucker-Davis Technologies). ECoG was recorded as described above (CyberAmp, Molecular Devices).

#### Ca^2+^-imaging

The craniotomy required for Ca^2+^-imaging was performed similarly as described above. During surgery, the surface of the cerebellar cortex was cleaned with extracellular solution composed of (in mM) 150 NaCl, 2.5 KCl, 2 CaCl_2_, 1 MgCl_2_ and 10 HEPES (pH 7.4, adjusted with NaOH). After the craniotomy, the mice were allowed to recover from isoflurane anesthesia for at least 30 min and were subsequently head-fixed in the recording setup and received a bolus-loading of the Ca^2+^ indicator Cal-520 (0.2 mM; AAT Bioquest, Sunnyvale, CA) (Stosiek et al., 2003; Tsutsumi et al., 2015). The dye was first dissolved with 10% w/V Pluronic F-127 in pure DMSO (Invitrogen) and diluted 20 times in the extracellular solution. The dye solution was pressure injected into the molecular layer (50–80 μm below the surface) at 0.35 bar for 5 min. Finally, the brain surface was covered with 2% agarose dissolved in saline (0.9% NaCl) in order to reduce motion artifacts and prevent dehydration.

Starting at least 30 min after dye injection, thus at least 1 hr after the end of isoflurane administration, *in vivo* two-photon Ca^2+^-imaging was performed of the molecular layer using a setup consisting of a titanium sapphire laser (Chameleon Ultra, Coherent, Santa Clara, CA, USA), a TriM Scope II system (LaVisionBioTec, Bielefeld, Germany) mounted on a BX51 microscope with a 20X 1.0 NA water immersion objective (Olympus, Tokyo, Japan) and a GaAsP photomultiplier detector (Hamamatsu, Iwata City, Japan). A typical recording sampled ~20 × 200 μm with a frame rate of ~50 Hz.

### Data analyses

#### Offline GSWD detection

The start and end of GSWD episodes and timing of ECoG “spikes” (i.e., negative ECoG peaks during episodes of GSWDs) were detected using a custom-written GSWD detection algorithm (LabVIEW, National Instruments, Austin, TX, USA) as described previously (Kros et al., 2015b). The program detects GSWD-episodes with a minimal duration of 1 s and a minimal interval between seizures of 1 s. Ictal periods were defined as the time from start to end of seizures and interictal periods refer to the time between 2 s after a seizure and 2 s before the next.

#### Detection of action potentials in extracellular recordings

Extracellular recordings were included if activity was well isolated for >100 s and action potential detection in extracellular traces was performed using threshold-based analyses with customized Matlab (Mathworks Inc. Natick, MA, USA) routines or the Matlab-based program SpikeTrain (Neurasmus BV, Erasmus MC Holding, Rotterdam, Netherlands). Complex spikes were separated from simple spikes using waveform-based cluster analyses and *post-hoc* visual inspection.

#### Detection of calcium events

After motion correction, the dendrites of Purkinje cells were identified using spatial independent component analysis. From each dendrite, the fluorescent signal was used to detect transients as described previously (De Gruijl et al., 2014).

#### GSWD-related firing pattern modulation

To assess whether Purkinje cells showed GSWD-modulated firing patterns, a custom-written algorithm in LabVIEW (National Instruments) was used as described previously (Kros et al., 2015b). Since all ECoG channels (M1 and S1) consistently showed SWDs simultaneously, we only used the M1 recording for GSWD analysis. GSWD-triggered rasterplots and peri-stimulus time histograms (PSTH) with a 10 ms bin width were created for both complex spike and simple spike firing if the total GSWD-episode length was at least 2 s. This allowed us to determine: (1) modulation amplitude: the amplitude difference between the peak and trough near *t* = 0 (corresponding to the spikes of the GSWDs); (2) modulation frequency: dominant frequency in the PSTH as determined with Fast Fourier Transforms (FFT); and (3) mean power at GSWD frequency: based on the average power between 6 and 9 Hz (GSWD frequency range) as determined with FFT. Due to the low complex spike frequency and the low total number of ictal complex spikes, complex spike-based PSTHs of GSWD-modulated cells often did not show a sinusoidal distribution like those seen for GSWD-modulated simple spike firing. Therefore, only modulation amplitude and modulation frequency were considered for complex spike activity. Subsequently, the interspike intervals used for this PSTH were randomly shuffled (500 times) and converted into new PSTHs in order to create normal distributions of modulation amplitude and mean power at GSWD frequency. Z-scores (Z = (X-μ)/σ) could now be calculated based on the real and shuffled data. Complex spike firing was identified as GSWD-modulated if: (1) The modulation amplitude was significantly higher than expected by chance (Z ≥ 1.96, *p* ≤ 0.05); and (2) the cell modulated at GSWD frequency (6–9 Hz). For simple spike activity, a third rule was added: the mean power at GSWD frequency was significantly higher than expected by chance (Z ≥ 1.96, *p* ≤ 0.05). Since all ictal simple spike firing that showed significant Z-scores of mean power at GSWD frequency also showed significantly higher modulation amplitudes, the former was used for further analyses. The term “Z-score” without specification refers to modulation amplitude for complex spike firing and to mean power at GSWD frequency for simple spike firing throughout the manuscript.

Phase differences between the ECoG-spike of GSWDs and the peak of Purkinje cell complex spike and simple spike activity were calculated using the time between 0 (aligned to the peak of the ECoG-spike) and the first peak in the PSTH divided by the median GSWD distance and multiplied by 360.

#### Firing pattern parameters

Firing pattern parameters were assessed using custom written LabVIEW (National Instruments) based programs described previously (Kros et al., 2015b) and calculating median climbing fiber pause, defined as the median time difference between a complex spike and the first consecutive simple spike, firing frequency, coefficient of variation (CV) of interspike intervals (ISIs) (σ_ISI_/μ_ISI_), CV2 *(2|ISI*_*n*+1_− *ISI*_*n*_*|/(ISI*_*n*+1_+ *ISI*_*n*_*))* (Holt et al., 1996) and burst index (number of action potentials within bursts/total number of action potentials) for which we defined “burst” as a sequence of ≥3 spikes within 100 ms. Firing patterns were specifically calculated for ictal and interictal periods. To make sure these interictal periods were really interictal and without abnormal pre-ictal or post-ictal activity, the 2 s before and after each seizure were excluded from analysis.

#### Synchronicity of Purkinje cell firing

To investigate the synchronicity of ictal Purkinje cell activity, cross-correlograms of pairs of simultaneously recorded Purkinje cells were calculated for both the complex spikes and the simple spikes using the xcorr function from Matlab's Signal Processing Toolbox (Mathworks). Cross-correlograms were normalized so that the autocorrelations at zero lag equaled 1. For the complex spike cross-correlograms, only Purkinje cells with a minimum of 10 ictal complex spikes were included in the analysis. The detected spike times of the complex spikes and simple spikes were binned before calculating the cross-correlogram of each Purkinje cell pair (10 ms bin size). Next we used the bootstrapping method in order to calculate the amount of synchronicity that would occur by chance. For this we randomly shuffled the interspike intervals of complex spikes and simple spikes of each Purkinje cell pair 500 times, creating 500 surrogate spiketrains for each Purkinje cell. From these bootstrapped spiketimes we calculated the cross-correlograms for the complex spikes and simple spikes as described above. Z-scores from the original cross-correlograms could now be calculated using the real and bootstrapped data by applying: Z = (X − μ)/σ where X indicates the values based on the original cross-correlogram, μ indicates the mean of the 500 bootstrapped cross-correlograms and σ indicates the standard deviation. Purkinje cell pairs were identified to be synchronously active if the cross-correlogram amplitude was significantly higher than expected by chance (Z > 1.96) at a lag of ~0 ms. In a similar fashion we also calculated the synchronicity for interictal Purkinje cell activity. The same analyses with the same criteria were applied to the imaging data using the timing of the calcium events.

#### Statistical analyses

We randomly assigned mice to either electrophysiological recordings or Ca^2+^-imaging experiments. Statistical differences in firing pattern parameters between independent groups of Purkinje cell recordings (GSWD-modulated, non-modulated and wild-type cells) were determined using MANOVA's with complex spike firing frequency, median climbing fiber pause, simple spike firing frequency, CV, CV2 and burst index as dependent variables and group as independent variable. If a MANOVA showed a significant result, *post-hoc* ANOVA's were used to assess contributions of individual firing pattern parameters with Bonferroni corrected *p*-values. Statistical differences in firing pattern parameters between ictal and interictal periods were tested with repeated measures ANOVA's with one “within-subjects” factor, i.e., time period, with 2 levels (ictal and interictal) and Bonferroni corrections. Differences in frequency of Ca^2+^-events between ictal and interictal periods were tested using a paired *t*-test.

A *p* ≤ 0.05 (α) was considered significant unless a Bonferroni correction was used; in that case a *p*-value of α/n was considered significant. Two-tailed testing was used for all statistical analyses and all were performed using SPSS 22.0 software (IBM Corporation, New York, USA). All data throughout the manuscript are represented as mean ± standard deviation (SD). *N* indicates the number of mice; *n* indicates the number of recordings. In the tables significant *p*-values are printed in bold.

## Results

### GSWD-related Purkinje cell firing patterns

**Figure 1:**
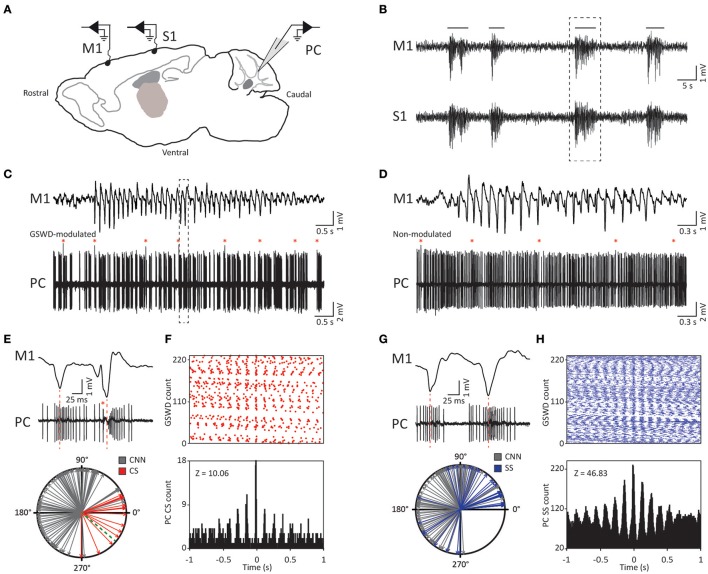
Purkinje cell complex and simple spike firing show GSWD-related modulation. **(A)** Schematic of recording conditions. **(B)** Example of typical GSWD-episodes in both primary motor (M1) and primary sensory (S1) cortices. Horizontal lines indicate GSWD-episodes. **(C)** Example of Purkinje cell spiking that aligns to boxed GSWD episode in **(B)**. Red asterisks indicate complex spikes. **(D)** Example of non-modulated Purkinje cell firing. **(E)** Example of the timing of a complex spike with respect to the GSWDs. This is activity of the same Purkinje cell as shown in **(C)** but during a different seizure. Red line aligns ECoG spike to Purkinje cell action potential firing. (Bottom panel) Compass plot of phase difference between modulated Purkinje cell complex spike firing (red) or cerebellar nuclei neuron action potential firing (gray) and ECoG spikes. Dashed green line refers to the onset of the ECoG spike (e.g., the start of the downward deflection in the ECoG). **(F)** Raster plot (top) and PSTH (bottom) for complex spike firing during nine GSWD-episodes in the example in C; *t* = 0 indicates each ECoG spike. **(G,H)** As in **(E,F)**, for GSWD-modulated simple spike firing of the cell shown in **(C)** [dashed box in **C** is magnified in **G** (top)]. The phase information of the cerebellar nuclei data (gray arrows in the compass plots in **E**,**G**) was previously published in Kros et al. (2015b) and is merely shown here for comparison with phase relations of Purkinje cell activity. PC, Purkinje cell; M1, Primary motor cortex; S1, Primary somatosensory cortex; CNN, Cerebellar nuclei neuron; CS, Complex Spikes; SS, Simple spikes; GSWD, Generalized spike and wave discharge.

To establish to what extent activity of the inferior olive and the cerebellar Purkinje cells contribute to the oscillatory activity of cerebellar nuclei neurons during absence seizures, we investigated temporal relations of complex spike and simple spike firing patterns with GSWDs. To this end, extracellular Purkinje cell activity was simultaneously recorded with primary motor and primary sensory ECoG in awake, head fixed *tottering* mice (Figure 1A). These ECoG recordings revealed GSWDs, which are characterized by negative deflections that occur at a mean frequency of 7.74 ± 1.01 Hz (*N* = 23). GSWD-episodes had a mean duration of 3.53 ± 1.12 s (*N* = 23), which is comparable to previously reported values in rodent models for epilepsy (Noebels and Sidman, 1979; Kandel and Buzsáki, 1993; Kros et al., 2015b).

**Figure 2:**
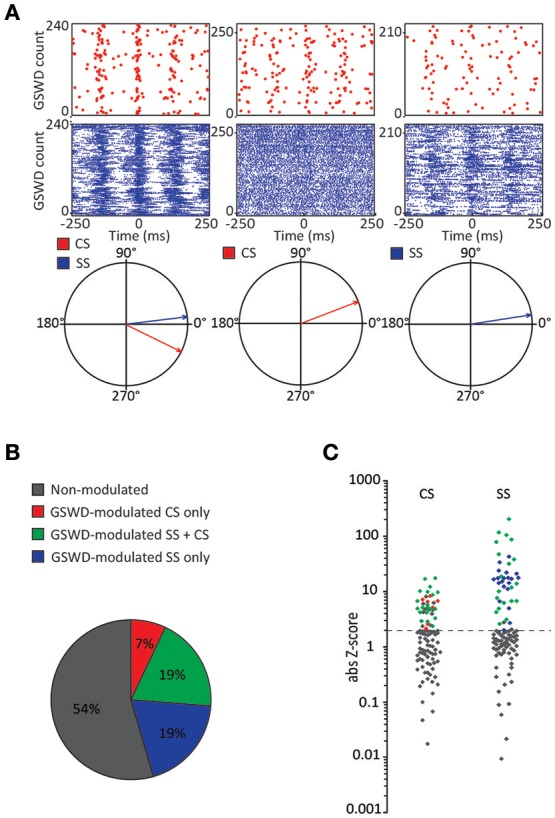
Co-occurrence of GSWD-modulated complex spike and simple spike firing **(A)** Examples of complex spike raster plots (red, top) simple spike raster plots (blue, middle) and compass phase plots (bottom) of Purkinje cells with GSWD-modulated complex spikes and simple spikes (left), GSWD-modulated complex spikes only (middle) and GSWD-modulated simple spike only (right). **(B)** Distribution of Purkinje cells showing GSWD-modulated complex spike firing only (red), GSWD-modulated simple spike firing only (blue), both simple spike and complex spike GSWD-modulated firing (green) and non-modulating (gray). **(C)** Distributions of FFT-based complex spike Z-scores (left) and modulation amplitude-based simple spike Z-scores (right) color coded according to the Purkinje cell distributions described in **(B)**. Dashed line corresponds to the cut-off Z-score of 1.96 (*p* < 0.05). Gray markers above this cut-off in the complex spike-based Z-score plot correspond to cells that showed significant modulation that was not in GSWD frequency range. GSWD, Generalized spike and wave discharge; CS, Complex spikes; SS, Simple spikes.

Similar to their main downstream targets, cerebellar nuclei neurons, a portion of Purkinje cells clearly showed GSWD-modulated complex spike or simple spike firing patterns (Figures 1C,D). Complex spike firing that was phase-locked to GSWDs occurred in 26% of Purkinje cells (Figures 1E,F). In these Purkinje cells, the peak of complex spike activity occurred almost exclusively during the ECoG spike, virtually all of them appeared after the onset of the ECoG spike (green dashed line in the compass plot in Figure 1E). Interestingly, this is the inverse pattern to that seen in cerebellar nuclei neurons (Figure 1E; cf. Kros et al., 2015b). GSWD-modulated simple spike firing tended to occur during the same phase of a GSWD as cerebellar nuclei firing (Figures 1G,H). These data indicate that during GSWD episodes the complex spike firing coincides with a pause in simple spike and cerebellar nuclei firing. Overall, almost half of the recorded Purkinje cells (47 out of 103) showed GSWD-modulated complex spike and/or simple spike firing. Of these modulated Purkinje cells, 15% showed complex spike modulation only, 43% showed both complex spike and simple spike modulation and in the remaining 43%, sole simple spike modulation was observed (Figures 2A–C).

**Figure 3:**
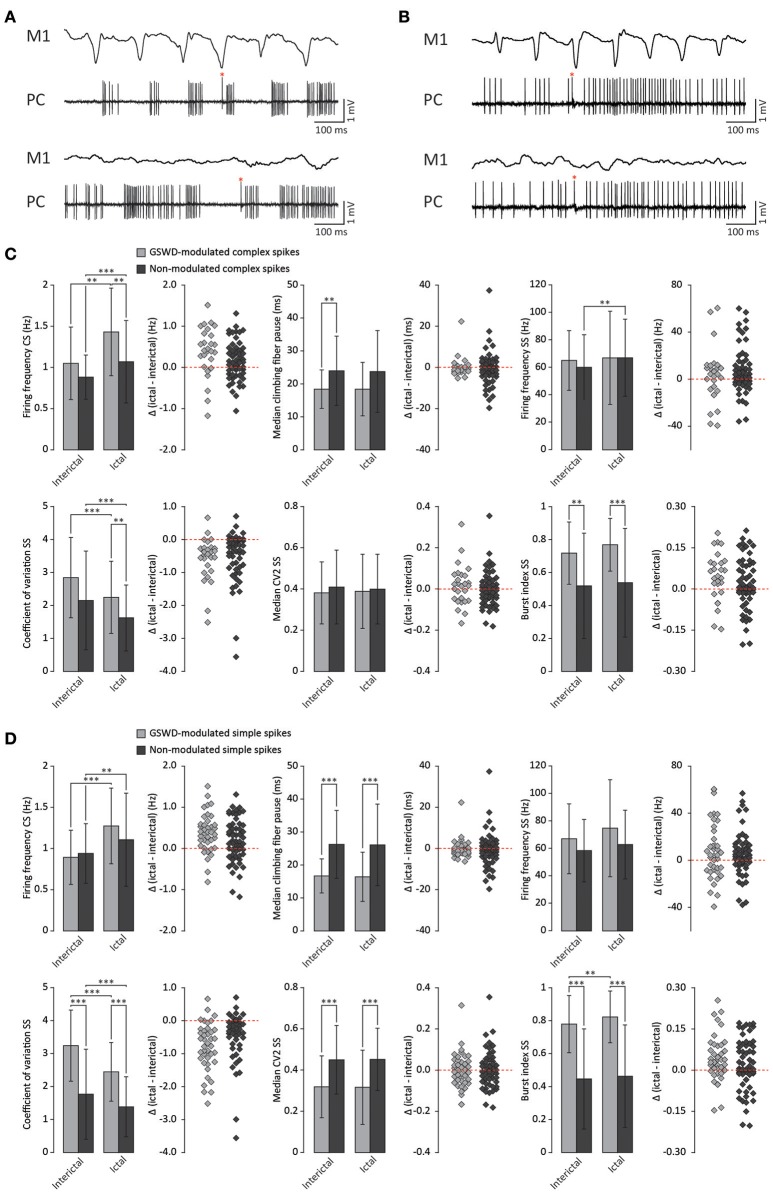
Differences in firing pattern parameters between GSWD-modulated and non-modulated Purkinje cells. **(A)** Representative example of ictal (top) and interictal (bottom) spiking from a single Purkinje cell showing GSWD-related modulation. **(B)** As in **(A)**, but for a non-modulated Purkinje cell. Red asterisks indicate complex spikes. **(C)** Spiking parameters for Purkinje cells showing GSWD-modulated (*n* = 27) and non-modulated (*n* = 76) complex spike firing. ^**^p < 0.01, ^***^p < 0.001 (Tables 1, 2). Error bars denote mean ± SD. Scatterplots correspond to the bar plots and represent for each firing pattern parameter the difference between ictal and interictal conditions per cell [i.e., delta (ictal-interictal)]. **(D)** As in **(C)**, but for Purkinje cells that showed GSWD-modulated (*n* = 40) and non-modulated (*n* = 63) simple spike firing. ^**^p < 0.01, ^***^p < 0.001 (Tables 3, 4). Error bars denote mean ± SD. PC, Purkinje cell; M1, Primary motor cortex.

We next investigated whether Purkinje cells that showed either GSWD-modulated complex spike or simple spike activity can be distinguished from non-modulating Purkinje cells (Figures 3A,B). Because firing patterns can be determined using various non-independent parameters that asses rate and regularity (Figures 3C,D), we first tested for differences in overall firing pattern using MANOVAs that, in case of significance, were followed by *post-hoc* ANOVAs. Firing patterns were calculated separately for interictal and ictal periods. This allowed us to compare modulated and non-modulated firing during GSWD-episodes and interictal periods separately. Additionally we could assess changes in firing between ictal and interictal periods. These analyses revealed that Purkinje cells showing GSWD-modulated complex spike firing indeed differed from cells that did not show complex spike modulation in both interictal (*p* < 0.05) and ictal (*p* < 0.01) firing patterns. More specifically, cells with GSWD-modulated complex spike firing showed a shorter interictal climbing fiber pause (*p* < 0.01), a higher simple spike burst index during both interictal (*p* < 0.01) and ictal periods (*p* ≤ 0.001), a higher ictal complex spike frequency (*p* < 0.01) and a higher coefficient of variation (CV) of ictal simple spike firing (*p* < 0.01) when compared to those cells that showed non-modulated firing (Figure 3C, Table 1). The same analyses based on modulated simple spike firing resulted in similar differences between modulated and non-modulated Purkinje cells (*p* < 0.001 both ictally and interictally; Figure 3D, Table 3). We also noticed that not only modulated Purkinje cells, but also non-modulated Purkinje cells (based on either complex spike or simple spike firing) showed a change in firing pattern when comparing ictal and inter-ictal firing patterns (Figures 3C,D, Tables 2, 4). Notably, our data show that during GSWDs, an inverse phase relationship exists between complex spike firing and both simple spike and cerebellar nuclei firing, and that the complex spike frequency increased significantly during seizures. This indicates that at least a proportion of inferior olivary neurons significantly changes activity during seizures. (see Figure 3, Tables 1–4 for all statistical outcomes for the individual firing pattern parameters).

**Table 1:** Differences in action Potential firing between Purkinje cells with non-modulated and GSWD-modulated complex spikes (Corresponding to Figure 3C).

**Tested variable**	**Compared groups**	***n***	***p*-value**	***F*-value**	**Statistical test**
Overall	Non-modulated CS interictal	76	**<0.05**	*F*_(6, 96)_ = 2.36	MANOVA (Pillai's trace)
	GSWD-modulated CS interictal	27			
Firing frequency CS	Non-modulated CS interictal	76	0.031	*F*_(1, 101)_ = 4.79	ANOVA (Bonferroni)
	GSWD-modulated CS interictal	27			
Median CF pause	Non-modulated CS interictal	76	**<0.01**	*F*_(1, 101)_ = 6.89	ANOVA (Bonferroni)
	GSWD-modulated CS interictal	27			
Firing frequency SS	Non-modulated CS interictal	76	0.406	*F*_(1, 101)_ = 0.70	ANOVA (Bonferroni)
	GSWD-modulated CS interictal	27			
Coefficient of Variation SS	Non-modulated CS interictal	76	0.034	*F*_(1, 101)_ = 4.62	ANOVA (Bonferroni)
	GSWD-modulated CS interictal	27			
CV2 SS	Non-modulated CS interictal	76	0.475	*F*_(1, 101)_ = 0.52	ANOVA (Bonferroni)
	GSWD-modulated CS interictal	27			
Burst index SS	Non-modulated CS interictal	76	**<0.01**	*F*_(1, 101)_ = 9.25	ANOVA (Bonferroni)
	GSWD-modulated CS interictal	27			
Overall	Non-modulated CS ictal	76	**<0.01**	*F*_(6, 96)_ = 9.73	MANOVA (Pillai's trace)
	GSWD-modulated CS ictal	27			
Firing frequency CS	Non-modulated CS ictal	76	**<0.01**	*F*_(1, 101)_ = 9.40	ANOVA (Bonferroni)
	GSWD-modulated CS ictal	27			
Median CF pause	Non-modulated CS ictal	76	0.041	*F*_(1, 101)_ = 4.27	ANOVA (Bonferroni)
	GSWD-modulated CS ictal	27			
Firing frequency SS	Non-modulated CS ictal	76	0.948	*F*_(1, 101)_ = 0.004	ANOVA (Bonferroni)
	GSWD-modulated CS ictal	27			
Coefficient of Variation SS	Non-modulated CS ictal	76	**<0.01**	*F*_(1, 101)_ = 7.64	ANOVA (Bonferroni)
	GSWD-modulated CS ictal	27			
CV2 SS	Non-modulated CS ictal	76	0.868	*F*_(1, 101)_ = 0.03	ANOVA (Bonferroni)
	GSWD-modulated CS ictal	27			
Burst index SS	Non-modulated CS ictal	76	≤ **0.001**	*F*_(1, 101)_ = 11.12	ANOVA (Bonferroni)
	GSWD-modulated CS ictal	27			

*CS, Complex spike; SS, Simple spike; CF, Climbing fiber; GSWD, Generalized spike and wave discharge*.

**Table 2:** Differences between ictal and interictal Purkinje cell action potential firing (Corresponding to Figure 3C).

**Tested variable**	**Compared groups**	***n***	***p*-value**	***F*-value**	**Statistical test**
Firing frequency CS	Non-modulated CS ictal	76	**<0.001**	*F*_(1, 75)_ = 15.55	Repeated measures ANOVA
	Non-modulated CS interictal				(Bonferroni)
Median CF pause	Non-modulated CS ictal	76	0.787	*F*_(1, 75)_ = 0.07	Repeated measures ANOVA
	Non-modulated CS interictal				(Bonferroni)
Firing frequency SS	Non-modulated CS ictal	76	**<0.01**	*F*_(1, 75)_ = 9.18	Repeated measures ANOVA
	Non-modulated CS interictal				(Bonferroni)
Coefficient of Variation SS	Non-modulated CS ictal	76	**<0.001**	*F*_(1, 75)_ = 36.13	Repeated measures ANOVA
	Non-modulated CS interictal				(Bonferroni)
CV2 SS	Non-modulated CS ictal	76	0.562	*F*_(1, 75)_ = 0.34	Repeated measures ANOVA
	Non-modulated CS interictal				(Bonferroni)
Burst index SS	Non-modulated CS ictal	76	0.040	*F*_(1, 75)_ = 4.38	Repeated measures ANOVA
	Non-modulated CS interictal				(Bonferroni)
Firing frequency CS	GSWD-modulated CS ictal	27	**<0.01**	*F*_(1, 26)_ = 10.95	Repeated measures ANOVA
	GSWD-modulated CS interictal				(Bonferroni)
Median CF pause	GSWD-modulated CS ictal	27	0.988	*F*_(1, 26)_ = 0.00	Repeated measures ANOVA
	GSWD-modulated CS interictal				(Bonferroni)
Firing frequency SS	GSWD-modulated CS ictal	27	0.559	*F*_(1, 26)_ = 0.35	Repeated measures ANOVA
	GSWD-modulated CS interictal				(Bonferroni)
Coefficient of Variation SS	GSWD-modulated CS ictal	27	**<0.001**	*F*_(1, 26)_ = 21.03	Repeated measures ANOVA
	GSWD-modulated CS interictal				(Bonferroni)
CV2 SS	GSWD-modulated CS ictal	27	0.409	*F*_(1, 26)_ = 0.70	Repeated measures ANOVA
	GSWD-modulated CS interictal				(Bonferroni)
Burst index SS	GSWD-modulated CS ictal	27	0.015	*F*_(1, 26)_ = 6.85	Repeated measures ANOVA
	GSWD-modulated CS interictal				(Bonferroni)

*CS, Complex spike; SS, Simple spike; CF, Climbing fiber; GSWD, Generalized spike and wave discharge*.

**Table 3:** Differences in action potential firing between Purkinje cells with non-modulated and GSWD-modulated simple spikes (Corresponding to Figure 3D).

**Tested variable**	**Compared groups**	***n***	***p*-value**	***F*-value**	**Statistical test**
Overall	Non-modulated SS interictal	63	**<0.001**	*F*_(6, 96)_ = 8.63	MANOVA (Pillai's trace)
	GSWD-modulated SS interictal	40			
Firing frequency CS	Non-modulated SS interictal	63	0.511	*F*_(1, 101)_ = 0.44	ANOVA (Bonferroni)
	GSWD-modulated SS interictal	40			
Median CF pause	Non-modulated SS interictal	63	**<0.001**	*F*_(1, 101)_ = 29.82	ANOVA (Bonferroni)
	GSWD-modulated SS interictal	40			
Firing frequency SS	Non-modulated SS interictal	63	0.078	*F*_(1, 101)_ = 3.18	ANOVA (Bonferroni)
	GSWD-modulated SS interictal	40			
Coefficient of Variation SS	Non-modulated SS interictal	63	**<0.001**	*F*_(1, 101)_ = 33.32	ANOVA (Bonferroni)
	GSWD-modulated SS interictal	40			
CV2 SS	Non-modulated SS interictal	63	**<0.001**	*F*_(1, 101)_ = 16.36	ANOVA (Bonferroni)
	GSWD-modulated SS interictal	40			
Burst index SS	Non-modulated SS interictal	63	**<0.001**	*F*_(1, 101)_ = 39.71	ANOVA (Bonferroni)
	GSWD-modulated SS interictal	40			
Overall	Non-modulated SS ictal	63	**<0.001**	*F*_(6, 96)_ = 9.73	MANOVA (Pillai's trace)
	GSWD-modulated SS ictal	40			
Firing frequency CS	Non-modulated SS ictal	63	0.120	*F*_(1, 101)_ = 2.46	ANOVA (Bonferroni)
	GSWD-modulated SS ictal	40			
Median CF pause	Non-modulated SS ictal	63	**<0.001**	*F*_(1, 101)_ = 19.89	ANOVA (Bonferroni)
	GSWD-modulated SS ictal	40			
Firing frequency SS	Non-modulated SS ictal	63	0.048	*F*_(1, 101)_ = 4.02	ANOVA (Bonferroni)
	GSWD-modulated SS ictal	40			
Coefficient of Variation SS	Non-modulated SS ictal	63	**<0.001**	*F*_(1, 101)_ = 33.81	ANOVA (Bonferroni)
	GSWD-modulated SS ictal	40			
CV2 SS	Non-modulated SS ictal	63	**<0.001**	*F*_(1, 101)_ = 16.90	ANOVA (Bonferroni)
	GSWD-modulated SS ictal	40			
Burst index SS	Non-modulated SS ictal	63	**<0.001**	*F*_(1, 101)_ = 45.92	ANOVA (Bonferroni)
	GSWD-modulated SS ictal	40			

*CS, Complex spike; SS, Simple spike; CF, Climbing fiber; GSWD, Generalized spike and wave discharge*.

**Table 4:** Differences between ictal and interictal Purkinje cell action potential firing (Corresponding to Figure 3D).

**Tested variable**	**Compared groups**	***n***	***p*-value**	***F*-value**	**Statistical test**
Firing frequency CS	Non-modulated SS ictal	63	**<0.01**	*F*_(1, 62)_ = 6.98	Repeated measures ANOVA
	Non-modulated SS interictal				(Bonferroni)
Median CF pause	Non-modulated SS ictal	63	0.896	*F*_(1, 62)_ = 0.02	Repeated measures ANOVA
	Non-modulated SS interictal				(Bonferroni)
Firing frequency SS	Non-modulated SS ictal	63	0.059	*F*_(1, 62)_ = 3.70	Repeated measures ANOVA
	Non-modulated SS interictal				(Bonferroni)
Coefficient of Variation SS	Non-modulated SS ictal	63	**<0.001**	*F*_(1, 62)_ = 18.57	Repeated measures ANOVA
	Non-modulated SS interictal				(Bonferroni)
CV2 SS	Non-modulated SS ictal	63	0.857	*F*_(1, 62)_ = 0.03	Repeated measures ANOVA
	Non-modulated SS interictal				(Bonferroni)
Burst index SS	Non-modulated SS ictal	63	0.143	*F*_(1, 62)_ = 2.20	Repeated measures ANOVA
	Non-modulated SS interictal				(Bonferroni)
Firing frequency CS	GSWD-modulated SS ictal	40	**<0.001**	*F*_(1, 39)_ = 26.89	Repeated measures ANOVA
	GSWD-modulated SS interictal				(Bonferroni)
Median CF pause	GSWD-modulated SS ictal	40	0.709	*F*_(1, 39)_ = 0.14	Repeated measures ANOVA
	GSWD-modulated SS interictal				(Bonferroni)
Firing frequency SS	GSWD-modulated SS ictal	40	0.049	*F*_(1, 39)_ = 4.15	Repeated measures ANOVA
	GSWD-modulated SS interictal				(Bonferroni)
Coefficient of Variation SS	GSWD-modulated SS ictal	40	**<0.001**	*F*_(1, 39)_ = 48.31	Repeated measures ANOVA
	GSWD-modulated SS interictal				(Bonferroni)
CV2 SS	GSWD-modulated SS ictal	40	0.857	*F*_(1, 39)_ = 0.03	Repeated measures ANOVA
	GSWD-modulated SS interictal				(Bonferroni)
Burst index SS	GSWD-modulated SS ictal	40	**<0.01**	*F*_(1, 39)_ = 10.78	Repeated measures ANOVA
	GSWD-modulated SS interictal				(Bonferroni)

*CS, Complex spike; SS, Simple spike; CF, Climbing fiber; GSWD, Generalized spike and wave discharge*.

**Figure 4:**
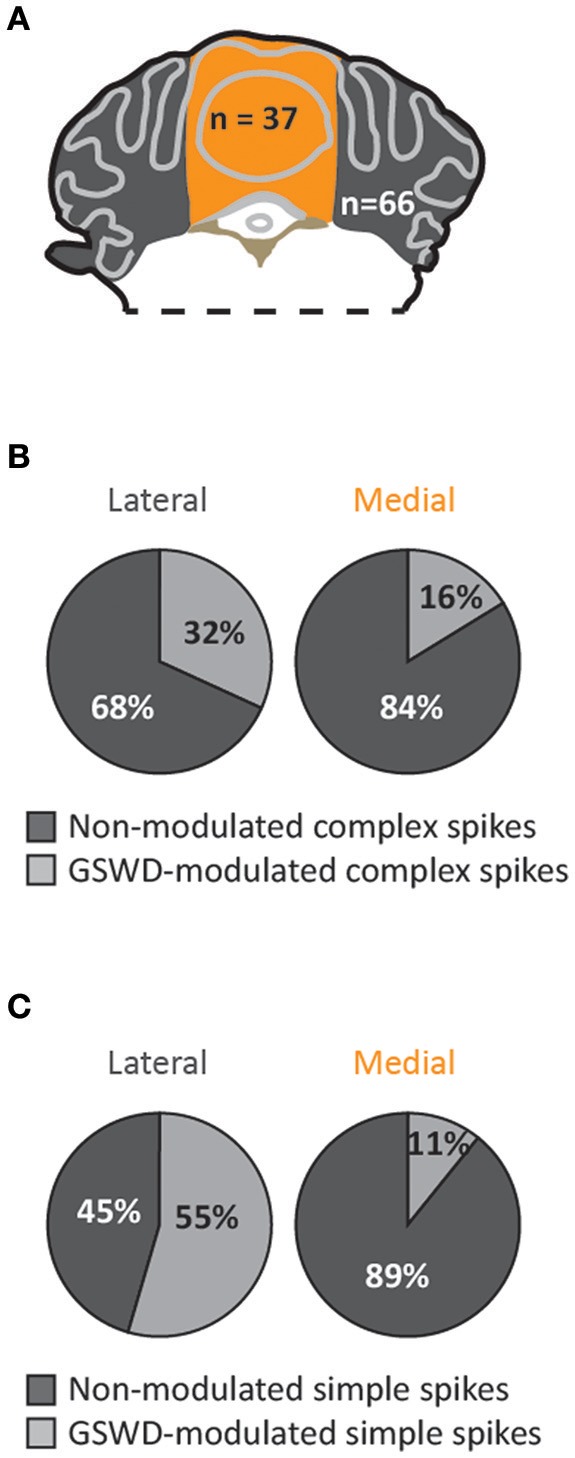
Purkinje cells showing GSWD-modulated complex spike firing are most prominently present in lateral parts. **(A)** Schematic representation of lateral cerebellar area (gray) and the vermis (yellow). **(B,C)** Proportion of Purkinje cells showing GSWD-modulated complex spikes **(B)** or simple spikes **(C)** in lateral (left) or medial (right) cerebellar areas.

When we assessed the location of GSWD-modulated and non-modulated Purkinje cells, we found that Purkinje cells that showed GSWD-modulated complex spikes are found more often in the lateral cerebellum (hemispheres) compared to the medial cerebellum (vermis) (Figures 4A,B). When looking at simple spike activity, this difference was even larger (Figure 4C). These data suggest that GSWD-modulated Purkinje cell firing patterns are more likely to be found in cerebellar hemispheres, possibly reflecting a cerebral cortical origin (Voogd et al., 2013).

### GSWD related Purkinje cell synchronicity: electrophysiology

**Figure 5:**
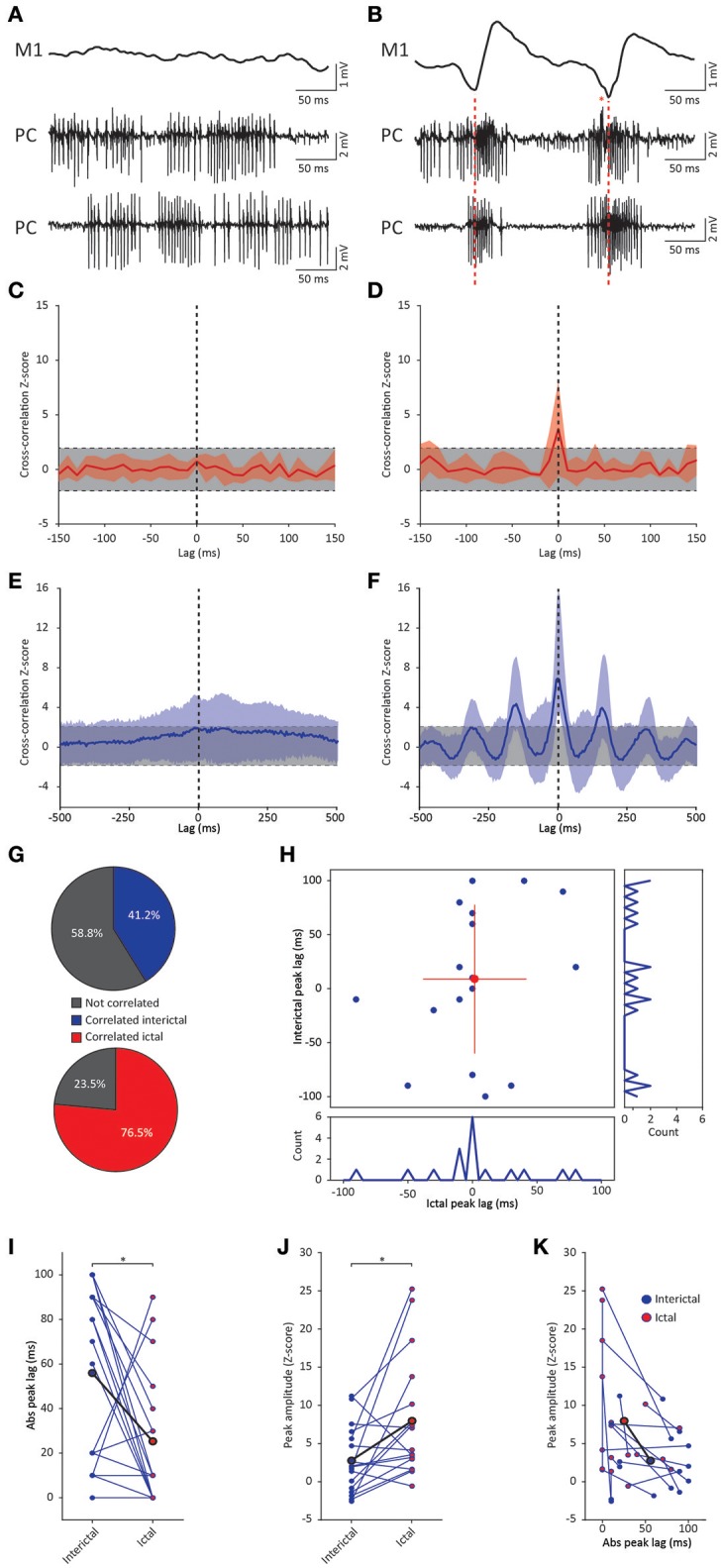
GSWD-related synchronicity in Purkinje cell complex spike and simple spike firing. **(A,B)** Example of interictal, non-synchronous Purkinje cell firing **(A)** and ictal GSWD-modulated, synchronous activity **(B)** of two simultaneously recorded Purkinje cells. Red dashed lines indicate ECoG-spikes. Red asterisks indicate complex spikes. **(C,D)** Average cross-correlation (red line) ± SD (shaded light red area) of complex spike firing of two cells during interictal periods **(C)** and GSWD episodes **(D)**. Strength of the correlation is expressed in Z-scores with respect to the cross-correlations of a bootstrapped distribution (gray bar, 500 x; see Material and Methods section). The vertical, black dashed line depicts a lag of 0 ms. **(E,F)** As in **(C,D)**, but the for average cross-correlation Z-scores ± *SD* (shaded light blue area) of simple spike firing. **(G)** Proportion of cell pairs that show a significant cross-correlation Z-score during interictal (blue, top graph) and ictal (red, bottom graph) periods. **(H)** Distribution of the peak lags in the individual cross-correlograms. Y-axis shows the interictal peak lag and the horizontal axis shows the ictal peak lag. PSTHs of interictal (right) and ictal (bottom) peak lags (bin size is 10 ms). **(I,J)** Differences in absolute peak lag **(I)** and peak amplitude **(J)** between interictal (blue dots) and ictal periods (red dots). Blue lines represent the individual cell pairs and the black line shows the average. ^*^p < 0.05 (Table 5). **(K)** Combined differences in absolute peak lag and peak amplitude between interictal (blue dots) and ictal periods (red dots). (*N* = 8, *n* = 17 pairs). PC, Purkinje cell; M1, Primary motor cortex.

The occurrence of robust GSWD related changes in complex spike activity suggests a potential inferior olivary role in seizures. We next aimed to further investigate how olivary firing is affected by thalamocortical network oscillations. Since each olivary neuron innervates ~7 Purkinje cells in the same sagittal plane and different parts of the inferior olive project to different cerebellar zones (Van der Want et al., 1989; De Zeeuw et al., 1998; Voogd and Ruigrok, 2004; Apps and Garwicz, 2005; Sugihara et al., 2009), simultaneously recorded activity from distant Purkinje cells can help elucidate the degree of synchronicity across different olivary zones. Additionally, given the extensive degree of convergence of Purkinje cell to cerebellar nuclei projections, not only the timing of Purkinje cell action potentials but also the synchronicity of the activity plays an important role in shaping cerebellar nuclei firing patterns. Therefore, assessing synchronicity of Purkinje cell activity by combining ECoG with multiple single-unit Purkinje cell recordings in awake *tottering* mice (Figures 5A,B) allowed us to both investigate GSWD related olivary activity and assess its potential contribution to oscillatory cerebellar output.

Cross-correlation analysis revealed no significantly increased interictal average complex spike synchronicity compared to a bootstrapped distribution (Figure 5C). This indicates that complex spikes recorded during interictal periods did not occur simultaneously. During ictal periods however, a significant cross-correlation was found with an average lag of 1.25 ± 24.75 ms (range −50–40 ms; *n* = 8) (Figure 5D). These data indicate that there was no indication of increased average complex spike synchronicity during interictal periods, but that during seizures distant Purkinje cell pairs do tend to fire complex spikes synchronously. The degree of synchronicity of electrophysiologically recorded Purkinje cell activity was not found to be preferentially dependent on relative distance on the mediolateral or the anteroposterior axis (data not shown). This suggests that distant inferior olive neurons show synchronous activity during seizures. Simultaneously occurring complex spikes are also likely to profoundly impact downstream cerebellar nuclei activity. Inducing synchrony in complex spike firing at a general scale has previously been shown to result in a pause followed by a burst of action potentials in cerebellar nuclei neurons (Hoebeek et al., 2010) and given the high degree of ictal synchrony in Purkinje cell firing, this is also likely to contribute to the burst-like firing pattern of many cerebellar nuclei neurons during GSWD-episodes (Kros et al., 2015b).

Cerebellar nuclei firing has also been shown to be subject to simple spike synchronicity (Gauck and Jaeger, 2000; De Zeeuw et al., 2008; Person and Raman, 2012). Whereas asynchronous simple spike activity has been shown to dampen cerebellar nuclei activity, increased synchronous simple spike firing entrained rather than inhibited cerebellar nuclei activity (Person and Raman, 2012). This would also be in line with the lack of phase difference between GSWD-related simple spike and cerebellar nuclei firing. We therefore performed the same cross-correlation analyses on these Purkinje cell pairs to assess simple spike synchrony. Similar to complex spike firing, average simple spike synchronicity was only increased during GSWD-episodes but not during interictal periods (Figures 5E,F). Seven out of 17 (41%) individual pairs showed significantly correlated interictal simple spike firing, which increased to 13 out of 17 (76.5%) during ictal periods (Figure 5G). Analyses of the timing of the peak cross-correlation shows that during GSWDs the peak lag is much closer to zero than during interictal periods (*p* < 0.05, Figures 5H,I, Table 5). The peak amplitude also showed a significant difference between ictal and interictal periods; during GSWDs, the cross-correlation was significantly increased (*p* < 0.05, Figure 5J, Table 5). The difference in lag between interictal and ictal periods was not predictive of a difference in amplitude (*p* = 0.405, Figure 5K, Table 5). Thus, the synchronicity and rhythmicity of Purkinje cell simple spike firing are stronger during ictal periods.

**Table 5:** Differences between ictal and interictal Purkinje cell synchronicity (Corresponding to Figure 5).

**Tested variable**	**Compared groups**	***n***	***p*-value**	***F* or *t*-value**	**Statistical test**
Absolute peak lag	Ictal	17	**<0.05**	*t*_(1, 16)_ = 6.13	Paired *t*-test
	Interictal				
Peak amplitude	Ictal	17	**<0.05**	*t*_(1, 16)_ = 7.23	Paired *t*-test
	Interictal				
Ictal—interictal	Delta lag	17	0.405	*t*_(1, 16)_ = 1.82	Linear regression
	Delta amplitude				

### GSWD related Purkinje cell synchronicity: Ca^2+^-imaging

**Figure 6:**
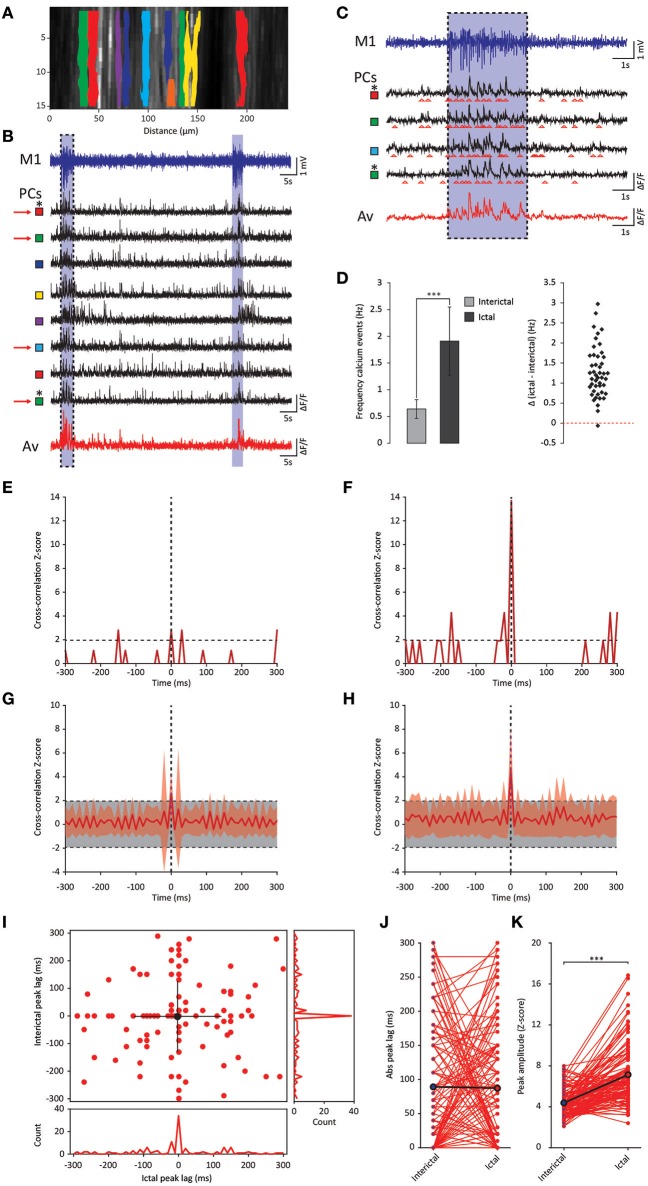
GSWD-related synchronicity of Ca^2+^-events in adjacent Purkinje cells. **(A)** Example of a window showing Purkinje cell dendrites and corresponding dendritic masks during two-photon Ca^2+^-imaging. Note that the y-axis is magnified and differs from the x-axis in scale. **(B)** Representative example of ECoG (blue top trace) and fluorescent traces recorded from various Purkinje cell dendrites (black traces) during interictal and ictal (light blue shades) periods. The bottom red trace represents the average Ca^2+^-signal of all black traces above. Colored squares next to the traces refer to the dendritic masks shown in **(A)**. **(C)** Magnification of the boxed data in **(B)** (marked with red arrows). **(D)** Difference in frequency of Ca^2+^-events of Purkinje cells (*n* = 46) between interictal and ictal periods (left). Scatterplots correspond to the bar plots and represent for each parameter the difference between ictal and interictal conditions per cell. ^***^p < 0.001 (Table 6). Error bars denote mean ± SD. **(E,F)** Example of a cross-correlogram of 2 Purkinje cells marked in **(B,C)** with asterisks during interictal **(E)** and ictal **(F)** periods. Strength of the correlation is expressed in Z-scores with respect to the cross-correlations of a bootstrapped distribution (horizontal black dashed line). The vertical black dashed line depicts a lag of 0 ms. **(G,H)** As in **(E,F)** but with the average cross-correlation Z-scores ± SD (shaded light red area) of the population (*n* = 119 pairs). **(I)** Distribution of peak lags in the individual cross-correlograms. Y-axis shows the interictal peak lag and the horizontal axis shows the ictal peak lag. PSTHs of interictal (right) and ictal (bottom) peak lags. **(J,K)** Differences in absolute peak lag **(J)** and peak amplitude **(K)** between interictal (blue dots) and ictal periods (red dots). Red lines represent the individual cell pairs and the black the average. ^*^p < 0.001 (Table 6). PC, Purkinje cell; M1, Primary motor cortex; Av, Average.

Whereas the multiple-unit recordings described above can be used to assess interictal and ictal complex spike synchrony of Purkinje cells positioned in different zones or lobules, we did not assess activity patterns of adjacent Purkinje cells, which are much more likely to get climbing fiber input from clusters of coupled olivary neurons (De Zeeuw et al., 2011). We therefore performed two-photon Ca^2+^-imaging of awake *tottering* mice while simultaneously recording the ECoG (Figure 6A). Results from these imaging experiments showed a marked increase in both the frequency and synchronicity of Ca^2+^-events (Figures 6B–H) in ictal periods when compared to interictal activity. The increase in both frequency (*p* < 0.001, Figure 6D, Table 6) and synchronicity (Figures 6F,H) confirmed our electrophysiological data (Figure 5). In these experiments we did, however, find significant complex spike cross-correlations during interictal periods (Figures 6E,G) occurring with a lag of ~0 ms (Figures 6I,J). In line with the data shown in Figure 4, ictal synchronicity in all Purkinje cell pairs was again significantly higher than that observed during interictal periods (*p* < 0.001, Figure 6K, Table 6). Moreover, this increase in ictal compared to interictal synchronicity remained highly significant even in the subset of Purkinje cell pairs that showed a significant cross-correlation Z-score at a lag of 0 ms (*p* < 0.001, Table 6). Synchronicity of Ca^2+^ events did not show a dependence on inter-dendritic distance during interictal periods [*t*_(1, 118)_ = 0.077, *R*^2^ = 0.00, *p* = 0.939] or ictal periods [*t*_(1, 118)_ = −1.105, *R*^2^ = 0.010, *p* = 0.271] as tested with linear regression analyses. Additionally the difference between the interictal and ictal cross-correlation peaks does not show any distance dependency [*t*_(1, 118)_ = −0.669, *R*^2^ = 0.004, *p* = 0.505].

**Table 6:** Differences between ictal and interictal Purkinje cell calcium events (Corresponding to Figure 6).

**Tested variable**	**Compared groups**	***n***	***p*-value**	***t*-value**	**Statistical test**
Calcium event frequency	Ictal	46	**<0.001**	*t*_(1, 45)_ = 13.50	Paired *t*-test
	Interictal				
Absolute peak lag	Ictal	120	0.849	*t*_(1, 120)_ = −0.19	Paired *t*-test
	Interictal				
Peak amplitude	Ictal	120	**<0.001**	*t*_(1, 120)_ = 3.73	Paired *t*-test
	Interictal				
Peak amplitude	Ictal with peak lag of 0 ms	14	**<0.001**	*t*_(1, 13)_ = 5.01	Paired *t*-test
	Interictal with peak lag of 0 ms				

## Discussion

The inferior olive has been implicated to play a role in generalized epilepsy (Harding and Boyd, 1991; Welsh et al., 1998; Saito et al., 2006). In the current study we aimed to investigate absence seizure related complex spike activity patterns with a particular focus on rhythmicity and synchronicity. Simultaneously recording activity in the cerebral cortex and the cerebellar cortex in awake, head-restrained homozygous *tottering* mice revealed pronounced GSWD-related changes in Purkinje cell firing. We show that complex spike and simple spike activity of a substantial subset of Purkinje cells is phase locked to GSWDs during seizures. Simultaneous recordings of ensembles of Purkinje cells, either by multiple single-unit recordings or Ca^2+^-imaging, showed a significant increase in complex spike activity and synchronicity in both distant and adjacent cells during ictal events, indicating profound and widespread changes in olivary firing during absence seizures.

### GSWD-related changes in olivary activity and synchronicity

Complex spike firing frequency, rhythmicity as well as synchronicity were all significantly increased during ictal episodes compared to inter-ictal periods, and the phase-locked complex spike rhythmicity was more prominent in the lateral than medial cerebellar cortex. These differences suggest that the GSWD-related activity changes in the inferior olive are induced in the thalamocortical network, rather than the other way around (see also Polack et al., 2007; Sorokin et al., 2017). The cerebral cortex projects to the principal olive (PO) and rostral medial accessory olive (rMAO) mainly via the mesodiencephalic junction (De Zeeuw et al., 1998). The PO and rMAO in turn provide climbing fibers to the lateral, but not the medial, cerebellum (De Zeeuw and Ten Brinke, 2015). If the inferior olivary cells would have induced or promoted the GSWDs in the thalamocortical network, because of the fact that the olivary cells also express the *Cacna1a* gene encoding the Ca_V_2.1-channels (Choi et al., 2010), one would expect to also observe prominent complex spike rhythmicity and synchronicity phase-locked to GSWDs in the medial cerebellar cortex, which receives its climbing fiber inputs from the caudal MAO and caudal dorsal accessory olive. Moreover, if one looks carefully at the precise moment when the complex spikes during the GSWD-episodes start to occur (dashed line in bottom panel of Figure 1E), one can see that the vast majority (i.e., ~90%) of the complex spikes occurs after the start of the falling phase of the spike of the GSWD, which reflects the onset of cortical burst-firing (Polack et al., 2007). Together, these data suggest that the increased rhythmicity and synchronicity are more likely to be the consequence rather than the cause of the GSWD.

One might also consider the possibility that the profound changes in olivary activity during seizures form a mechanism to compensate for the over-oscillating brain during seizures. Indeed, the capability of olivary neurons to dampen oscillations is supported by recent work in Dr. Sillitoe's lab (White and Sillitoe, 2017), who showed that blocking olivary output increases rhythmicity in the motor output, precisely at the frequency (6–9 Hz) that can be optimally dampened by olivary activity (Llinás and Yarom, 1981; Khosrovani et al., 2007; Llinás, 2009). Neurons in the inferior olive have unique properties to support such a function; their dendrites are electrically coupled by gap junctions and endowed with conductances that promote oscillations, together enhancing synchrony and rhythmicity in complex spike firing when the system requires such modification (Blenkinsop and Lang, 2006; De Zeeuw et al., 2011). The concept of the inferior olive and olivocerebellar system as a compensator for potentially pathological oscillations is also in line with the finding that children with dentato-olivary dysplasia can suffer from intractable seizures (Harding and Boyd, 1991; Saito et al., 2006). When the olivary oscillatory activity cannot be properly controlled anymore by the GABAergic feedback from the cerebellar nuclei (De Zeeuw et al., 1998; Svensson et al., 2006; Bazzigaluppi et al., 2012; Lefler et al., 2014), compensatory effects could be ameliorated. Likewise, the threshold for pharmacologically induced seizures is reduced in rats following chemical ablation of the inferior olive (Anderson et al., 1987, 1988, 1990), supporting the same mechanism.

### GSWD-modulated Purkinje cell simple spikes

Much like their downstream targets (Kros et al., 2015b) and in line with previous results in an inbred rat strain characterized with absence epilepsy (“WAG/Rij” strain) (Kandel and Buzsáki, 1993), about half of the recorded Purkinje cells showed an ictal simple spike firing pattern that was phase-locked to GSWDs. GSWD-modulated simple spike activity showed a similar phase-relation to GSWDs as cerebellar nuclei neurons; bursts of activity during the ECoG waves interspersed with pauses coinciding with ECoG spikes. In contrast, complex spike activity predominantly showed the inverse phase-relation. Simple spike and complex spike firing have been shown to often be reciprocally modulated; periodic decreases in simple spike firing often coincide with increases in complex spike firing and vice versa. This antiphasic temporal relation between complex spike and simple spike firing becomes most prominently evident during sensorimotor control (Graf et al., 1988; De Zeeuw et al., 1995; Yakhnitsa and Barmack, 2006; Badura et al., 2013), but may also explain the antiphasic relation in GSWD modulation.

The firing patterns of *tottering* Purkinje cells have been described in detail using both *in vitro* and *in vivo* recording techniques (Hoebeek et al., 2005; Walter et al., 2006). One of the most pronounced aberrations is the increased irregularity of simple spike firing, which correlates to impaired motor behavior (Hoebeek et al., 2005) and has been attributed to the decreased Ca^2+^-influx and the subsequently decreased Ca^2+^-dependent K^+^-channel activity that disrupt the intrinsic pacemaking activity (Walter et al., 2006). Our current analyses of ictal and interictal firing patterns confirmed the aberrant Purkinje cell firing pattern in awake *tottering* mice, but also showed that GSWD-modulated cells can be distinguished from non-modulated cells even by analyzing inter-ictal firing patterns. This is particularly noteworthy, since we previously showed that also the interictal activity pattern of cerebellar nuclei neurons was predictive for their firing pattern during GSWD-episodes (Kros et al., 2015b). Together these data suggest that different populations of cerebellar neurons may have a different level of susceptibility to be modulated by thalamocortical oscillations.

### Potential role of Purkinje cell activity in dampening ictal cerebellar nuclei oscillations

The cerebellar nuclei have been utilized to effectively stop seizure activity in mice (Kros et al., 2015b) and men (Šramka et al., 1976; Šramka and Chkhenkeli, 1990; Chkhenkeli et al., 2004). During GSWD-episodes, a substantial subset of cerebellar nuclei neurons has been shown to fire in burst-like GSWD-modulated patterns. Pharmacological or optogenetic manipulation of cerebellar nuclei neuronal firing can potently and bidirectionally affect seizure occurrence (Kros et al., 2015b). Cerebellar nuclei neurons receive input from climbing and mossy fiber collaterals as well as local interneurons, but the majority of their synaptic inputs originates from Purkinje cells (De Zeeuw and Berrebi, 1995; Person and Raman, 2012). Although the impact of Purkinje cell firing and synchronicity has been addressed at both modeling and experimental level (for references see below), it remains to be elucidated how GSWD-modulated Purkinje cell activity and synchronicity precisely affect cerebellar nuclei neuron firing and thereby the cerebellar output during absence seizures.

Given the inhibitory effect of Purkinje cell activity on cerebellar nuclei neuronal firing (Ito et al., 1964, 1970), one would expect an antiphasic relation between Purkinje cell and cerebellar nuclei activity. Yet such a reciprocal relation was only found with complex spike but not simple spike firing. Large scale synchronization of olivary activity has been shown to evoke a pause followed by increased firing in cerebellar nuclei neurons (Hoebeek et al., 2010) resembling the pattern of bursts and pauses seen during GSWD-episodes. Although the characteristics of these rebound responses have been particularly well described (Aizenman and Linden, 2000; Gauck et al., 2001; Gauck and Jaeger, 2003; Molineux et al., 2006; Pugh and Raman, 2006; Feng and Jaeger, 2008; Alviña et al., 2009; Zheng and Raman, 2009; Hoebeek et al., 2010; Sangrey and Jaeger, 2010; Engbers et al., 2011; Feng et al., 2013), the existence of rebound responses in the cerebellar nuclei is however a heavily debated subject (Aksenov et al., 2004; Holdefer et al., 2005; Alviña et al., 2008; De Zeeuw et al., 2008, 2011; Hoebeek et al., 2008, 2010; Person and Raman, 2012; Heck et al., 2013; Witter et al., 2013; ten Brinke et al., 2015). Yet, the large scale synchronization of olivary neurons during these pathological network oscillations may resemble artificial olivary stimulation rather than the smaller scale synchronization associated with normal sensorimotor functions. Although recent evidence indicates that the direct olivary input to cerebellar nuclei neurons via climbing fiber collaterals provide considerable excitatory drive (Lu et al., 2016; Najac and Raman, 2017) our data represented in Figure 1 indicate that during GSWDs the excitation from olivary axon collaterals would arrive mostly during the pause in cerebellar nuclei firing, further highlighting the importance of Purkinje cell firing in directing GSWD-related cerebellar activity.

Simple spike activity has also been shown to affect cerebellar nuclei activity in a synchrony-dependent manner; rather than suppressing cerebellar nuclei activity, as has been shown for asynchronous simple spike patterns, synchronous simple spike activity tends to entrain cerebellar nuclei neuron firing (Person and Raman, 2012). Additionally, the impact of mossy fiber collaterals on cerebellar nuclei activity has been shown to depend on the degree of synchrony of their Purkinje cell inputs with increased levels of excitation upon increased levels of Purkinje cell synchronicity (Wu and Raman, 2017). We found that in a large subset of recorded Purkinje cell pairs, ictal simple spike synchronicity was significantly increased. This synchronicity likely explains the co-occurrence of bursts of simple spike activity and cerebellar nuclei activity. Given that the silencing of cerebellar nuclei firing results in a detrimental increase in GSWD occurrence (Kros et al., 2015b), our current data on Purkinje cell firing implicate that not only complex spikes, but also simple spike firing could in principle dampen seizures by fine-regulating GSWD-modulated firing of cerebellar nuclei neurons.

## Author contributions

LK, OE, LWJB, CD, and FH conceived and designed the study. LK, SL, OE, PM, and FH performed all recordings; LK, SL, LB, LWJB and FH designed and performed the analyses; CD and FH contributed financial support; LK, SL, OE, PM, LB, LWJB, CD, and FH co-wrote the manuscript.

### Conflict of interest statement

The authors declare that the research was conducted in the absence of any commercial or financial relationships that could be construed as a potential conflict of interest.
