# Corrigendum: Synchronicity and Rhythmicity of Purkinje Cell Firing during Generalized Spike-and-Wave Discharges in a Natural Mouse Model of Absence Epilepsy

**DOI:** 10.3389/fncel.2017.00369

**Published:** 2017-11-14

**Authors:** Lieke Kros, Sander Lindeman, Oscar H. J. Eelkman Rooda, Pavithra Murugesan, Lorenzo Bina, Laurens W. J. Bosman, Chris I. De Zeeuw, Freek E. Hoebeek

**Affiliations:** ^1^Department of Neuroscience, Erasmus MC, Rotterdam, Netherlands; ^2^Department of Neurosurgery, Erasmus MC, Rotterdam, Netherlands; ^3^Netherlands Institute for Neuroscience, Royal Dutch Academy for Arts and Sciences, Amsterdam, Netherlands

**Keywords:** epilepsy, cerebellum, inferior olive, tottering, oscillations

In the original article, there was an error in the title.

A correction has been made: “Synchronicity and Rhythmicity of Purkinje Cell Firing during Generalized Spike-and-Wave Discharges in a Natural Mouse Model of Absence Epilepsy” instead of “Synchronicity and Rhythmicity of Purkinje Cell Firing during Generalized Spike-and-Wave Discharges in a Natural Mouse Model of Absence Epilepsy Complex Spike Synchronicity during GSWDs”.

The authors apologize for this error and state that this does not change the scientific conclusions of the article in any way.

The original article has been updated.

## Conflict of interest statement

The authors declare that the research was conducted in the absence of any commercial or financial relationships that could be construed as a potential conflict of interest.

